# Teacher's Emotional Support and Math Performance: The Chain Mediating Effect of Academic Self-Efficacy and Math Behavioral Engagement

**DOI:** 10.3389/fpsyg.2021.651608

**Published:** 2021-09-03

**Authors:** Yanfei Yang, Guangzheng Li, Zhanguo Su, Yuan Yuan

**Affiliations:** ^1^Faculty of Human Development, Universiti Pendidikan Sultan Idris, Tanjong Malim, Malaysia; ^2^School of Education Science, Jiangsu Normal University, Xuzhou, China; ^3^Faculty of Physical Education, HuaiNan Normal University, Huainan, China; ^4^School of Teacher Education, Heze University, Heze, China

**Keywords:** teacher's emotional support, academic self-efficacy, math behavioral engagement, chain mediating, math performance

## Abstract

Positive teacher-student interaction can exert a positive influence on student engagement and math performance. As an important part of teacher-student interaction, emotional support of a teacher plays an indispensable role in the math performance of junior middle school and elementary school students. This study aimed to explore the effects of teacher's emotional support on math performance, and examine the mediating role of academic self-efficacy and math behavioral engagement. A total of 1,294 students in grades 3–5 and 7–8 from 14 junior middle and primary schools in China took part in the web-based survey. Results showed the following: (1) academic self-efficacy mediated the relationship between teacher's emotional support and math performance of Chinese primary and middle school boys and girls; math behavioral engagement mediated the relationship between teacher's emotional support and math performance of Chinese primary and middle school boys and girls; (2) The relationship between teacher's emotional support and math performance of Chinese junior middle school boys and girls was mediated by the chain of academic self-efficacy and math behavioral engagement.

## Introduction

Studies have found that a variety of factors, such as gender stereotypes, emotions, and attitudes, affect the mathematics performance of students (Hattie, 2009; Peixoto et al., 2017; Moè, 2018). Already in the first years of primary school, girls self-reported to be less able than boys in mathematics (Fredericks and Eccles, 2002; Moè, 2018) and identify with mathematics less than boys (Cvencek et al., 2011). Furthermore, teachers (Li, 1999) and parents (Tomasetto et al., 2011) believe boys to be more skilled than girls in mathematics. These negative stereotypes about mathematics might prompt girls to engage less in mathematics (Eddy and Brownell, 2016). The elementary school girls experience higher levels of math anxiety and boredom, and less self-efficacy compared with the boys (Lichtenfeld et al., 2012; Lohbeck et al., 2016).

To address this issue, researchers guided by self-determination theory (SDT; Deci and Ryan, 2000), a broad framework for the study and explanation of human motivation and personality, have shown evidence of the role played by the teacher support (Ryan and Deci, 2009; Núñez and León, 2015). Gender makes differences in the effects of teacher-student relationship quality (McGrath and Van Bergen, 2015). The gender socialization hypothesis (Ewing and Taylor, 2009) suggests that girls will be more affected by the level of support they receive from teachers, based on the finding that girls value close relationships more highly than do boys. Contrary to the academic risk hypothesis, low levels of teacher warmth are more detrimental to the academic achievement of elementary school boys than girls (Hamre and Pianta, 2001; Spilt et al., 2012). Individual perception of warmth and care of a teacher refers to the teacher's emotional support (Hamre and Pianta, 2007).

Prior research has found that both student-report and teacher-report of teacher emotional support decline across early adolescence (Wu and Hughes, 2015). In adolescence, an emotionally supportive teacher-student relationship may communicate acceptance, confidence in the ability of youth, respect for the autonomy of youth, and learning motivation and engagement of students will increase accordingly (Davis, 2006; Gregory et al., 2016). Consistent with this view, middle school students who perceive supportive relationships with teachers report more positive changes in school adjustment, learning emotions, and learning behaviors (Sakiz et al., 2012; Wang and Dishion, 2012; Chen et al., 2018). The research results of Kashy-Rosenbaum et al. (2018) show that the emotional support of tutors has a positive impact on individual academic performance. A longitudinal study confirmed that the support of teachers can predict the academic self-efficacy of the students (Jungert and Koestner, 2015), and when students perceived positive emotional support from teachers, it could promote their learning fun, learning self-efficacy, and learning engagement (Liu et al., 2018). The purpose of this study was to explore whether the relationship between emotional support of teachers and math academic performance could be explained separately by boys and girls of primary and junior middle school. Based on these studies, we examined whether academic self-efficacy and math behavioral engagement of students played a mediating role between teacher's emotional support and math performance. This study further explores whether there are gender differences in the influence of teacher emotional support on the academic performance of students and whether there are differences in different periods.

## Theoretical Basis and Hypothesis

### Perceived Teachers Emotional Support and Math Performance

Based on the theory of self-determination, perceived teacher emotional support refers to that an individual feels an emotional connection with the teacher in the classroom, and the teacher is interested and sensitive to his needs and responds positively and enthusiastically to him (Pianta et al., 2008). Students have a sense of security in the classroom, which allows them to explore new things and expand their experiences. Such emotional connections can promote learning motivation (Downer et al., 2010). The more encouragement a child receives from teachers in early childhood (from kindergarten through third grade above) and the more harmonious the teacher-student relationship is, the more likely is that the student achieves good academic and social development (Silver et al., 2005). In high-quality teacher-student interaction, the emotional support of teachers for children is crucial. Hamre and Pianta (2005) found that improving the emotional support of teachers can effectively reduce the risk of first-grade students dropping out of school. The subjects who also had inattention, low academic levels, and easy to have behavioral problems were randomly divided into two groups and were given different levels of emotional support from teachers, respectively. The results showed that the group with the lower emotional support of teachers showed low academic achievement and easy to conflict with teachers. Many previous studies have shown a strong relationship between social support (teacher support, peer support, and parent support) and academic achievement among middle and high school students (Rosenfeld et al., 2000). In a representative large sample study, in Maslow's hierarchy of needs, the need for affective and belonging precedes the need for knowledge. It is difficult for students to succeed academically when they are emotionally unfulfilled. Therefore, exploring the relationship between teacher's emotional support and math performance of junior middle school and primary school students can enhance the understanding of the teacher-student relationship, help them better adapt to school life, and thus lay a certain foundation for their social adaptation in adulthood.

### The Mediating Effect of Academic Self-Efficacy

Academic self-efficacy refers to expectation and judgment of the individuals that they are capable of completing specific learning tasks (Bandura, 1986). This study focuses on the embodiment of self-efficacy in the field of mathematics learning, which refers to the judgment of students and evaluation of their ability to complete mathematics learning tasks and achieve learning goals (Hackett and Betz, 1989). Research on gender stereotype threat has demonstrated that the motivation, engagement, and performance of girl students can suffer a negative stereotype, such as men outperforming women on math tests (Rosenthal et al., 2007). Students believe that they are capable of completing learning tasks, which is the foundation of academic achievement and personal success (McWilliams, 2014). Self-efficacy also predicted the math performance of primary and middle school students (Usher et al., 2019). The higher the level of self-efficacy of the student, the easier it is to achieve better academic performance (Komarraju and Nadler, 2013; MacPhee et al., 2013).

According to the self-determination theory proposed by Ryan and Deci (2000), the emotional support behavior of teachers satisfies student needs for competence and belonging (Jin and Wang, 2019). When these needs of students are met, it is conducive to the development of self-efficacy. Perceived teacher support can enhance the sense of self-efficacy in the individuals (Scott and Walczak, 2009) and trigger the internal and external motivation of students (Deci and Ryan, 2004). Students with more perceived teacher support have higher academic self-efficacy (Kim et al., 2018).

### The Mediating Effect of Behavioral Engagement

Although the predictive effect of teacher's emotional support on math performance has been supported by many studies (Hamre and Pianta, 2005; Chen et al., 2018), the research on its internal influence mechanism is still relatively limited.

In this study, behavioral engagement refers to a process from students simply completing mathematical academic tasks and observing discipline to participating in mathematical learning activities in math class, which is a description of the degree of involvement in mathematics learning (Fredricks et al., 2004). Finn (1993) found that student behavioral engagement can predict performance on standardized achievement tests. The positive prediction effects of student behavioral engagement on student achievement and student dropout rate (Fredricks et al., 2004). Positive classroom interaction between teachers and students is very important to improve the behavioral engagement of students (Cooper, 2014). A study by Lee (2014) on 3,268 middle school students from 121 middle schools in the United States showed that learning engagement can significantly predict the academic achievement of middle school students.

As environmental variables, supportive factors from teachers can significantly affect the learning engagement of students (Brewster and Bowen, 2004). A study on middle school students in Hong Kong showed that teacher support was stronger than parental support and peer support in predicting the academic engagement and academic achievement of the students (Chen, 2005). As a kind of independent support “choice” provided by teachers can positively predict motivation, interest and cognitive, and behavioral and emotional engagement of the students in learning (Flowerday and Schraw, 2000, 2003; Flowerday et al., 2004). Meanwhile, existing studies have also found that emotional support from the teacher plays an important role in enhancing motivation, participation, cooperation, and emotional well-being of the students in learning (Meyer and Turner, 2007).

### The Chain Mediating Role of Academic Self-Efficacy and Behavior Engagement

Perceived teacher support indirectly affects primary school math learning engagement of students through academic self-efficacy (Liu et al., 2018). Students can feel the care, trust, and respect of the teacher for them (Roth and Weinstock, 2013), which can stimulate positive emotions of the students, thus generate positive self-evaluation and further enhance their sense of academic self-efficacy. Self-efficacy directly affects the way of thinking, behavior, and emotions (Goetz et al., 2010). The Self-efficacy beliefs of the Secondary school student related to learning and performance in math significantly predicted their behavioral and affective engagement in math positively while predicting their behavioral and affective disaffection negatively (Ozkal, 2019).

**Figure 1:**
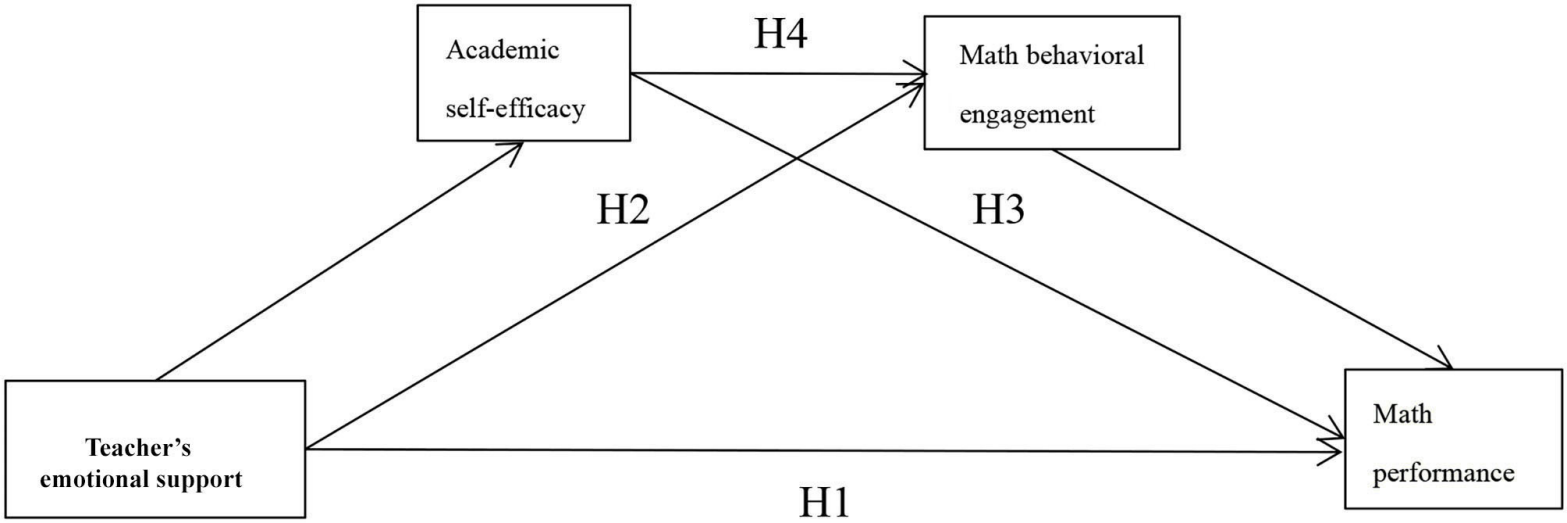
Hypothesized model.

According to social cognitive theory and self-determination theory, environment, personal factors (such as cognition, emotion, and motivation), and behavior interact. The emotional support of a teacher, as an environmental factor, can enhance individual self-efficacy (Scott and Walczak, 2009), show stronger learning vitality, and focus on their learning tasks (Jin and Wang, 2019). Figure 1 shows the hypothesized model.

We hypothesize the following:

H1: Teacher's emotional support is positively correlated with math performance.
H2: Academic self-efficacy plays a mediating role in the relationship between the teacher's emotional support and math performance.
H3: Behavioral engagement plays a mediating role in the relationship between the teacher's emotional support and math performance.
H4: Academic self-efficacy and behavioral engagement play a chain-mediated role in the relationship between the teacher's emotional support and math performance.

## Materials and Methods

### Participants

The convenience sampling method was adopted to select students from grades 3 to 5 and grades 7 to 8 of middle and primary schools in Henan and Gansu provinces of China, as the subjects for group testing. In total, 1,294 valid questionnaires were collected, with an effective rate of 91.5%. Among them, there were 657 boys (50.77%), 637 girls (49.23%), 233 students (18.01%) in the third grade, 219 students (16.92%) in the fourth grade, 285 students (22.02%) in the fifth grade, 223 students (17.23%) in the seventh grade, and 334 students (25.81%) in the eighth grade, with an average age of 12.05 ± 1.83 years old.

### Measures

#### Teacher's Emotional Support

The teacher's emotional support scale was used by Ertesvåg and Havik (2021). It consists of four items assessing emotional supportive behaviors of the teachers to be rated on a 4-points scale from 0 = Strongly disagree to 3 = Strongly agree (e.g., “The teachers are like my good friends”). The empirical factor analysis showed that the fit was good, and the indicators were as follows: *x*^2^*/df* = 2.026, RMSEA = 0.028, CFI = 0.999, TLI = 0.997. Cronbach's alpha values were 0.83.

#### Behavioral Engagement

The behavioral engagement scale which was originally developed by Wang et al. (2016) was used in the Chinese validation by Liu et al. (2018). It consists of eight items assessing the behavioral engagement of students in math to be rated on a 5-points scale from 1 = completely disagrees to 5 = completely agrees (e.g., “I completed my math homework on time”). After the empirical factor analysis, one item whose load was lower than 0.642 was deleted, and then the confirmatory factor analysis was conducted again, and the final behavioral engagement scale included seven items. After revision, confirmatory factor analysis of behavioral engagement showed good fit, and the indicators were as follows: *x*^2^*/df* = 4.056, RMSEA = 0.049, CFI = 0.987, and TLI = 0.979. The scale α = 0.78.

#### Academic Self-Efficacy

The academic self-efficacy scale was used in the Chinese validation by Lee et al. (2010). It consists of nine items assessing math self-efficacy of the students to be rated on a 5-points scale from 1 = not at all true to 5 = very true of me (e.g., “Compared with others in math class, I think I am a good student”). The empirical factor analysis showed that the fit was good, and the indicators were as follows: *x*^2^*/df* = 4.463, RMSEA = 0.052, CFI = 0.984, and TLI = 0.974. Cronbach's alpha value is 0.881.

#### Math Performance

In this study, math final exam results of the children are selected as the indicator of math performance. Studies have shown that the results of math courses can effectively represent the academic performance of Chinese children (Ding et al., 2012). In the process of data analysis, the math scores of students in different schools and different grades were converted into standard scores, and the scores obtained were finally used to calculate the math scores.

#### Demographic Variables

This study controlled for demographic variables, such as student age and grade, and ruled out possible effects of teacher's emotional support and math performance.

### Data Analysis

Our data analyses were conducted using SPSS 20.0, and the SPSS macro PROCESS (Hayes, 2013), and Amos 24.0. First, descriptive data were obtained using SPSS 20.0, and then Pearson's correlations were calculated to assess the correlations between the variables. Second, in accordance with Anderson and Gerbing (1988), we performed a two-step procedure to analyze the mediation effects. We first used two measurement models to test whether each latent variable could be well-represented by its indicators. We next determined whether the results from the measurement model were satisfactory; the two structural models could be tested using maximum likelihood (ML) estimation in the AMOS 24.0 program. Although the χ^2^ statistic is often reported, because of its dependence on sample size (Gessaroli and De Champlain, 1996), other indicators are commonly used to determine how well the model fits. According to Hu and Bentler (1999), the model fits well when CFI > 0.90, TLI > 0.90, and RMSEA <0.06.

Finally, the multi-mediation analyses were all conducted using the PROCESS macro in SPSS 20.0 (Preacher and Hayes, 2004; Hayes, 2013). The number of bootstrap samples for the bias-corrected bootstrap confidence intervals was 5,000.

## Results

### Common Method Deviation Test

Data collected by the self-report method may have a common method deviation, and Harman single factor test was adopted in this study (Podsakoff et al., 2003). It was found that there were seven factors with eigenvalues >1, which explained 61.861% variation. The variance explained by the first factor was 35.859%, less than the 40% threshold. Therefore, the common method deviation of data in this study is not serious.

### Confirmatory Factor Analysis

To test the discriminative validity of the variables in this study, confirmatory factor analysis was conducted for each variable using the AMOS 22.0 software. The results in Table 1 show that, compared with the single-factor model, two-factor model and three-factor model, the four-factor model adopted in this study is the most appropriate. The combination effect is ideal, and the fitting indexes of the four factors model all reach the standard, and the fitting degree of the model is good.

**Table 1:** Confirmatory factor analysis results of variable discriminant validity.

**Model**	***x^**2**^***	**df**	***x^**2**^/*df**	**CFI**	**GFI**	**TLI**	**IFI**	**NFI**	**RMSEA**
Four-factor model	894.950	182	4.917	0.938	0.935	0.929	0.939	0.924	0.055
Three-factor model	1235.700	182	6.790	0.909	0.909	0.895	0.909	0.895	0.067
Two-factor model	1286.235	179	7.186	0.904	0.908	0.888	0.905	0.891	0.069
Single-factor model	1243.897	179	6.949	0.908	0.902	0.892	0.908	0.895	0.068

*Four-factor model: emotional support of a perceived teacher, academic self-efficacy, math behavioral engagement, and academic performance. Three-factor model: teacher's emotional support + academic self-efficacy, math behavioral engagement, and academic performance. Two-factor model: teacher's emotional support + academic self-efficacy + math behavioral engagement and academic performance. Single-factor model: teacher's emotional support + academic self-efficacy + math behavioral engagement + academic performance*.

### Descriptive Statistics and Correlations

In this study, the subjects were divided into four groups: primary school boys, primary school girls, junior middle school boys, and junior middle school girls; and descriptive analysis was performed, respectively. Tables 2, 3 present the descriptive results of each variable and the correlation coefficients among the variables. The results showed that teacher's emotional support was positively correlated with academic self-efficacy, math behavioral engagement, and math performance. Academic self-efficacy is positively correlated with math behavioral engagement and math performance. There is a positive correlation between math behavioral engagement and math performance. This is consistent with the results of previous studies.

**Table 2:** Means, SD, and intercorrelations (primary school boys and girls).

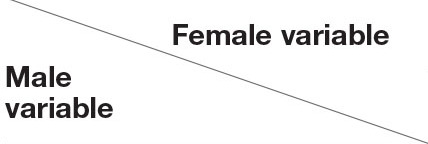	**Correlations**			
**variable**	**1**	**2**	**3**	**4**
1. Teacher's emotional support	1	0.574^**^	0.490^**^	0.235^**^
2. Academic self-efficacy	0.439^**^	1	0.629^**^	0.388^**^
3. Math behavioral engagement	0.406^**^	0.522^**^	1	0.315^**^
4. Math performance	0.208^**^	0.371^**^	0.282^**^	1
Mean	2.367	3.975	3.954	0.036
SD	0.580	0.662	0.703	0.920
Skewness	−0.926	−0.419	−0.447	−1.062
Kurtosis	0.570	−0.251	−0.353	0.664

*N = 737.*

** *P < 0.01*.

**Table 3:** Means, SD, and intercorrelations (junior middle school boys and girls).

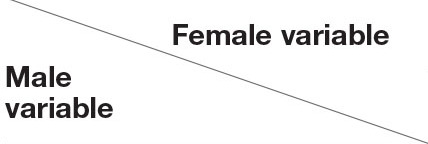	**Correlations**			
**variable**	**1**	**2**	**3**	**4**
1. Teacher's emotional support	1	0.522^**^	0.472^**^	0.277^**^
2. Academic self-efficacy	0.469^^**^^	1	0.587^**^	0.442^**^
3. Math behavioral engagement	0.394^**^	0.636^**^	1	0.406^**^
4. Math performance	0.187^**^	0.432^**^	0.403^**^	1
Mean	2.101	3.697	3.777	−0.010
SD	0.640	0.676	0.690	0.901
Skewness	−0.556	−0.213	−0.205	−0.541
Kurtosis	−0.104	−0.185	−0.541	0.035

*N = 557.*

** *P < 0.01*.

### Structural Equation Model Analyses

All subjects were divided into primary school boys, primary school girls, junior middle school boys, and junior middle school girls for structural equation model analysis.

#### Primary School Boys

First, the main effect was tested, with the teacher's emotional support as an independent variable and math performance as the dependent variable to construct the structural equation model 1.1. The fitting index of model 1.1 meets the requirements (*x*^2^*/*df = 2.164, CFI = 0.992, GFI = 0.991, TLI = 0.979, IFI = 0.992, NFI = 0.985, and RMSEA = 0.055); thus, the model fit is good. The main effect test results show that the teacher's emotional support positively affects math performance (B = 0.208, *p* < 0.001), and H1 is supported.

Second, models 1.2 and 1.3 were established with academic self-efficacy and math behavioral engagement as single mediators. The results show that the model fits well (Model 1.2: *x*^2^*/*df = 2.213, CFI = 0.959, GFI = 0.948, TLI = 0.946, IFI = 0.960, NFI = 0.928, and RMSEA = 0.056; Model 1.3: *x*^2^*/*df = 1.973, CFI = 0.965, GFI = 0.960, TLI = 0.954, IFI = 0.965, NFI = 0.931, and RMSEA = 0.050). Through process V3.5, the bootstrap method was used to repeat the sampling 5,000 times to test the mediating effect. The mediating effect of academic self-efficacy was 0.1507, with a 95% *CI* [0.0940, 0.2116], excluding 0, based on the assumption that H2 is verified. The mediating effect of math behavioral engagement is 0.0954, with 95% *CI* [0.0455, 0.1495], excluding 0, based on the assumption H3 is verified.

Finally, the chain multiple mediation effect was tested. A correlation was observed between the two mediator variables in academic self-efficacy and math behavioral engagement. The study assumes that the two variables play a mediating role in the impact of teacher's emotional support on math performance. Therefore, Hayes' multiple mediation method was used to test the mediating effect. According to process V3.5, the 95% *CI* of the mediating effect was estimated by extracting 5,000 bootstrap samples, and the chain multi-mediation effect of academic self-efficacy and math behavioral engagement was tested significantly. Teacher's emotional support → academic self-efficacy → math performance mediating effect is 0.1293, 95% *CI* is [0.0752, 0.1895], excluding 0, and mediating effect is significant. Teacher's emotional support → math behavioral engagement → math performance mediating effect is not significant. Teacher's emotional support → academic self-efficacy → math behavioral engagement → math performance chain multi-mediating effect is not significant, and H4 is not verified.

#### Primary School Girls

First, the main effect was tested, with the teacher's emotional support as an independent variable and math performance as the dependent variable to construct the structural equation model 2.1. The fitting index of model 2.1 meets the requirements (*x*^2^*/*df = 1.697, CFI = 0.992, GFI = 0.996, TLI = 0.985, IFI = 0.997, NFI = 0.993, and RMSEA = 0.044); thus, the model fit is good. The main effect test results show that teacher's emotional support positively affects math performance (B = 0.235, *p* < 0.001), and H1 is supported.

Second, models 2.2 and 2.3 were established with academic self-efficacy and math behavioral engagement as single mediators. The results show that the model fits well (Model 2.2: *x*^2^*/*df = 2.175, CFI = 0.960, GFI = 0.944, TLI = 0.947, IFI = 0.961, NFI = 0.929, and RMSEA = 0.058; Model 2.3: *x*^2^*/*df = 2.058, CFI = 0.966, GFI = 0.956, TLI = 0.955, IFI = 0.967, NFI = 0.937, and RMSEA = 0.055). Through process V3.5, the bootstrap method was used to repeat the sampling 5,000 times to test the mediating effect. The mediating effect of academic self-efficacy was 0.2164, with 95% *CI* [0.1342, 0.3046], excluding 0, based on the assumption that H2 is verified. The mediating effect of math behavioral engagement is 0.1290, with 95% *CI* [0.0670, 0.2010], excluding 0, based on the assumption H3 is verified.

Finally, the chain multiple mediation effect was tested. A correlation was observed between the two mediator variables in academic self-efficacy and math behavioral engagement. The study assumes that the two variables play a mediating role in the impact of teacher's emotional support on math performance. Therefore, Hayes' multiple mediation method was used to test the mediating effect. According to process V3.5, the 95% *CI* of the mediating effect was estimated by extracting 5,000 bootstrap samples, and the chain multi-mediation effect of academic self-efficacy and math behavioral engagement was tested significantly. Teacher's emotional support → academic self-efficacy → math performance mediating effect is 0.1811, 95% *CI* is [0.0952, 0.2719], excluding 0, and mediating effect is significant. Teacher's emotional support → math behavioral engagement → math performance mediating effect is not significant. Teacher's emotional support → academic self-efficacy → math behavioral engagement → math performance chain multi-mediating effect is not significant, and H4 is not verified.

#### Junior Middle School Boys

First, the main effect was tested, with the teacher's emotional support as an independent variable and math performance as the dependent variable to construct the structural equation model 3.1. The fitting index of model 3.1 meets the requirements (*x*^2^*/*df = 1.610, CFI = 0.996, GFI = 0.993, TLI = 0.988, IFI = 0.997, NFI = 0.991, and RMSEA = 0.047); thus, the model fit is good. The main effect test results show that teacher's emotional support positively affects math performance (*B* = 0.187, *p* < 0.01), and H1 is supported.

**Figure 2:**
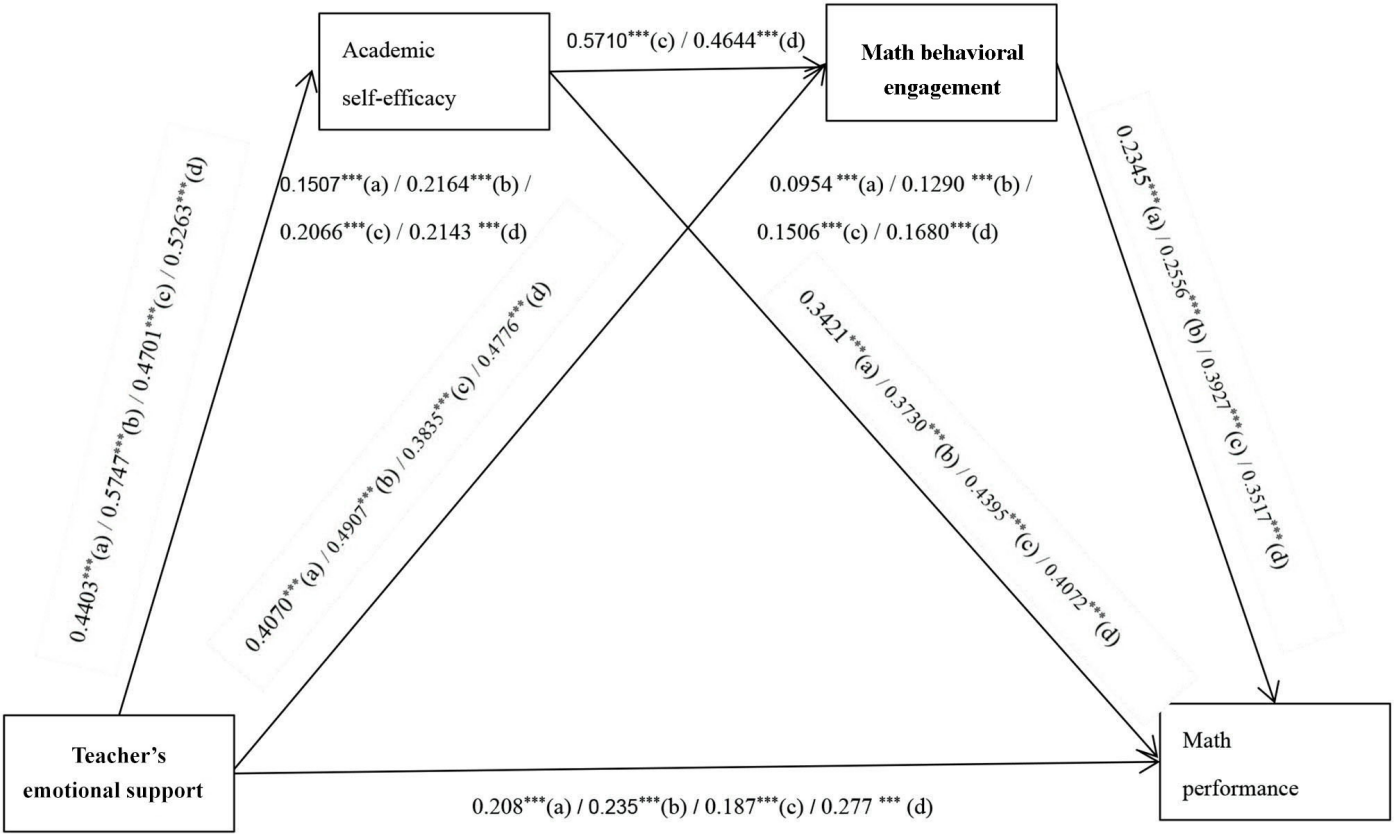
The standardized path coefficients in model testing. a = primary school boys; b = primary school girls; c = junior middle school boys; d = junior middle school girls.

Second, models 3.2 and 3.3 were established with academic self-efficacy and math behavioral engagement as single mediators. The results show that the model fits well (Model 3.2: *x*^2^*/*df = 1.817, CFI = 0.969, GFI = 0.956, TLI = 0.958, IFI = 0.970, NFI = 0.935, and RMSEA = 0.055; Model 3.3: *x*^2^*/*df = 1.948, CFI = 0.968, GFI = 0.936, TLI = 0.957, IFI = 0.968, NFI = 0.936, and RMSEA = 0.059). Through process V3.5, the bootstrap method was used to repeat the sampling 5,000 times to test the mediating effect. The results are shown in Figure 2. The mediating effect of academic self-efficacy was 0.2066, with 95% *CI* [0.1373, 0.2806], excluding 0, based on the assumption that H2 was verified. The mediating effect of math behavioral engagement is 0.1506, with 95% *CI* [0.0929, 0.2219], excluding 0, based on the assumption H3 is verified.

Finally, the chain multiple mediation effect was tested. A correlation was observed between the two mediator variables in academic self-efficacy and math behavioral engagement. The study assumes that the two variables play a mediating role in the impact of teacher's emotional support on math performance. Therefore, Hayes' multiple mediation method was used to test the mediating effect. According to process V3.5, the 95% *CI* of the mediating effect was estimated by extracting 5,000 bootstrap samples, and the chain multi-mediation effect of academic self-efficacy and math behavioral engagement was tested significantly. The results are shown in Table 4. Teacher's emotional support → academic self-efficacy → academic performance mediating effect is 0.1465, 95% *CI* is [0.0726, 0.2297], excluding 0, and mediating effect is significant. Teacher's emotional support → math behavioral engagement → academic performance, the mediating effect is not significant. Teacher's emotional support → academic self-efficacy → math behavioral engagement → academic performance chain multi-mediating effect is 0.0601, 95% *CI* [0.0173, 0.1063], excluding 0, indicating that academic self-efficacy and math behavioral engagement are between teacher's emotional support and academic performance, and H4 is verified.

**Table 4:** Bootstrap analyses of the significance of mediation.

	**Effect**	**Model pathways**	**Standardized effect value**	**SE**	**95% CI mean effect (lower and upper)**		**Effect of the amount**
**Junior middle school boys**							
	Direct effect	1. TES-MP	−0.0647	0.0870	−0.2359	0.1065	
	Intermediary effect	2. TES-ASE-MP	0.1465	0.0404	0.0719	0.2317	63.04%
		3. TES-MBE-MP	0.0257	0.0163	−0.0014	0.0625	
		4. TES-ASE-MBE-MP	0.0601	0.0230	0.0193	0.1097	25.86%
**Junior middle school girls**							
	Direct effect	1. TES-MP	0.0103	0.0908	−0.1685	0.1891	
	Intermediary effect	2. TES-ASE-MP	0.1609	0.0375	0.0887	0.2354	60.67%
		3. TES-MBE-MP	0.0509	0.0191	0.0180	0.0932	19.19%
		4. TES-ASE-MBE-MP	0.0534	0.0176	0.0213	0.0910	20.14%

*TES, teacher's emotional support; ASE, academic self-efficacy; MBE, math behavioral engagement; MP, math performance*.

#### Junior Middle School Girls

First, the main effect was tested, with the teacher's emotional support as an independent variable and math performance as the dependent variable to construct the structural equation model 4.1. The fitting index of model 4.1 meets the requirements (*x*^2^*/*df = 1.648, CFI = 0.993, GFI = 0.989, TLI = 0.985, IFI = 0.993, NFI = 0.981, and RMSEA = 0.048); thus, the model fit is good. The main effect test results show that teacher's emotional support positively affects math performance (B = 0.277, *p* < 0.001), and H1 is supported.

Second, models 4.2 and 4.3 were established with academic self-efficacy and math behavioral engagement as single mediators. The results show that the model fits well (Model 4.2: *x*^2^*/*df = 1.808, CFI = 0.965, GFI = 0.940, TLI = 0.953, IFI = 0.966, NFI = 0.926, and RMSEA = 0.054; Model 4.3: *x*^2^*/*df = 1.760, CFI = 0.972, GFI = 0.954, TLI = 0.963, IFI = 0.963, NFI = 0.937, and RMSEA = 0.052). Through process V3.5, the bootstrap method was used to repeat the sampling 5,000 times to test the mediating effect. The results are shown in Figure 2. The mediating effect of academic self-efficacy was 0.2143, with 95% *CI* [0.1470, 0.2842], excluding 0, based on the assumption that H2 was verified. The mediating effect of math behavioral engagement is 0.1680, with 95% *CI* [0.1091, 0.2318], excluding 0, based on the assumption H3 is verified.

Finally, the chain multiple mediation effect was tested. A correlation was observed between the two mediator variables in academic self-efficacy and math behavioral engagement. The study assumes that the two variables play a mediating role in the impact of teacher's emotional support on math performance. Therefore, Hayes' multiple mediation method was used to test the mediating effect. According to process V3.5, the 95% *CI* of the mediating effect was estimated by extracting 5,000 bootstrap samples, and the chain multi-mediation effect of academic self-efficacy and math behavioral engagement was tested significantly. The results are shown in Table 4. Teacher's emotional support → academic self-efficacy → academic performance mediating effect is 0.1609, 95% *CI* is [0.0896, 0.2356], excluding 0, and mediating effect is significant. Teacher's emotional support → math behavioral engagement → academic performance, the mediating effect is 0.0509, the 95% *CI* is [0.0176, 0.0925], excluding 0, and the mediating effect is significant. Teacher's emotional support → academic self-efficacy → math behavioral engagement → academic performance chain multi-mediating effect is 0.0534, 95% *CI* [0.0211, 0.0893], excluding 0, indicating that academic self-efficacy and math behavioral engagement are between teacher's emotional support and academic performance, and H4 is verified.

## Discussion

### Direct Relations

This study investigated the ways by which teacher's emotional support, academic self-efficacy, and math behavioral engagement affected the math performance of Chinese primary and middle school students. The results showed that teacher's emotional support could directly affect math performance of Chinese primary and middle school boys and girls, which was consistent with the study hypothesis H1 and previous research findings (Kashy-Rosenbaum et al., 2018). According to the ecosystem theory (Bronfenbrenner, 2009), school is the micro-system that has the closest influence on the development of students besides the family environment. As an important part of the school microsystem, the interaction between teachers and students will affect the academic performance and behavior of the student. The emotional support of the teacher satisfies the psychological needs of the student and stimulates learning motivation in the student. They are more willing to invest time and energy in learning tasks, and the more likely they are to achieve academic success.

### Mediated Role

The test results show that academic self-efficacy and math behavioral engagement play an intermediary role in the teacher's emotional support and math performance of Chinese primary and middle school boys and girls, respectively. Hypothesis 2 and 3 are verified. This is consistent with the results of previous studies. Emotional support of teacher behavior satisfies the needs of the student for competence and belonging (Jin and Wang, 2019). Self-determination theory (Ryan and Deci, 2000) believes that when these needs of students are met, it is conducive to the development of self-efficacy. The higher the level of self-efficacy of the student, the easier it is to achieve better academic performance (Komarraju and Nadler, 2013; MacPhee et al., 2013). Positive classroom interaction between teachers and students is very important to improve the behavioral engagement of the students (Cooper, 2014). As an important aspect of classroom interaction, the emotional support of the teacher plays an important role in enhancing the learning engagement of the student (Liu et al., 2018), and Lee (2014) found that middle school behavioral engagement of students can predict the performance of standardized achievement tests.

### The Chain Mediating Role

Academic self-efficacy enhanced the degree of math behavioral engagement and played a continuous intermediary role in the impact of teacher's emotional support on the math performance of Chinese junior middle school boys and girls. Hypothesis 4 is verified. This is consistent with the results of previous studies. Students can feel the care, trust, and respect of the teacher for them (Roth and Weinstock, 2013), which can stimulate positive emotions of the students, thus generate positive self-evaluation and further enhance their sense of academic self-efficacy. The increase of self-efficacy has a direct impact on the engagement of the student in learning behavior (Goetz et al., 2010), learning engagement can significantly predict academic achievement (Lee, 2014).

### Significance and Limitations of the Study

This study reveals the important role of teacher's emotional support, academic self-efficacy, and math behavioral engagement in math performance, and explores the chain mediating role of academic self-efficacy and math behavioral engagement. The strength of this study is to help broaden the knowledge about the contextual and individual motivational variables that can influence mathematical achievement. Therefore, the study addresses an important area and provides some new information/data. The results show the importance of the style and behavior of the teacher, which is of guiding significance for improving student engagement and math performance as well as education and teaching of teachers. The social system, school management, and teaching activities should be considered to promote teachers to adopt a supportive style of teaching.

But there are several weaknesses. Some statistical model indicators do not fit very well, probably because the data were collected by online channels. It is a cross-sectional study as it lacks longitudinal data and cannot accurately infer the causal relationship between variables. In the follow-up, it will investigate whether the relationship between teacher's emotional support, academic self-efficacy, math behavioral engagement, and math performance will change over time.

## Conclusion

The study explored the impact mechanism of the influence of teacher's emotional support on math performance based on social cognitive theory. The structural equation model was used to simultaneously test the individual and continuous mediation roles of academic self-efficacy and math behavioral engagement and verify the level of academic self-efficacy and behavioral engagement of the student in mathematics learning. The chain-based multi-mediating role in the relationship provides a new path toward considering the impact of teacher's emotional support on the math performance of intermediary mechanisms.

The empirical research shows the following results: (1) Main effect test. The results show a positive relationship between the teacher's emotional support and the math performance of Chinese primary and middle school boys and girls. (2) Intermediary effect test. The test results show that academic self-efficacy and math behavioral engagement play an intermediary role in the teacher's emotional support and math performance of Chinese primary and middle school boys and girls, respectively. Academic self-efficacy enhanced the degree of math behavioral engagement and played a continuous intermediary role in the impact of teacher's emotional support on the math performance of Chinese junior middle school boys and girls.

## Data Availability Statement

The original contributions presented in the study are included in the article/supplementary material, further inquiries can be directed to the corresponding authors.

## Ethics Statement

The studies involving human participants were reviewed and approved by Universiti Pendidikan Sultan Idris. Written informed consent to participate in this study was provided by the participants' legal guardian/next of kin.

## Author Contributions

GL: contributed to the conception of the study. YYu: contributed significantly to analysis and manuscript preparation. YYa: performed the data analyses and wrote the manuscript. ZS: performed the data analyses. All authors are participants in the data collection and analysis, writing, and revising the manuscript.

## Conflict of Interest

The authors declare that the research was conducted in the absence of any commercial or financial relationships that could be construed as a potential conflict of interest.

## Publisher's Note

All claims expressed in this article are solely those of the authors and do not necessarily represent those of their affiliated organizations, or those of the publisher, the editors and the reviewers. Any product that may be evaluated in this article, or claim that may be made by its manufacturer, is not guaranteed or endorsed by the publisher.
